# Supplementary material for: Functional specialization and interaction in the amygdala-hippocampus circuit during working memory processing
Source: Nat Commun. 2023 May 22;14:2921. doi: 10.1038/s41467-023-38571-w (PMC10203226; doi:10.1038/s41467-023-38571-w)
Supplement: Supplementary file 1 — Supplementary Information [file 41467_2023_38571_MOESM1_ESM.pdf]

Supplementary information

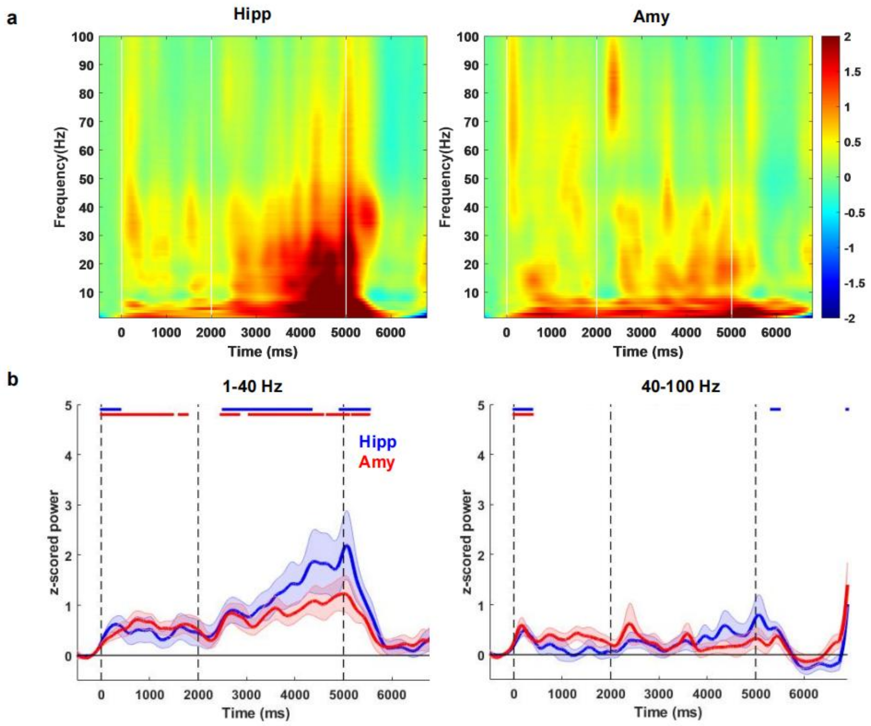

Figure S1 Time-frequency analyses of *z*-scored power within the amygdala and the hippocampus during WM. a The time-frequency plot of *z*-scored power in the hippocampus (left) and the amygdala (right). Warmer color denotes higher *z*-scored power. **b** Averaged *z*-scored power (± s.e.m. shown as shading around the mean trace) across 1-40 Hz (left) and 40-100 Hz (right) at each time point in the hippocampus (blue) and the amygdala (red). Blue and red lines at the top denote the time point with an activity significantly above zero (two-sided cluster-based permutation test, $p < 0.05$).

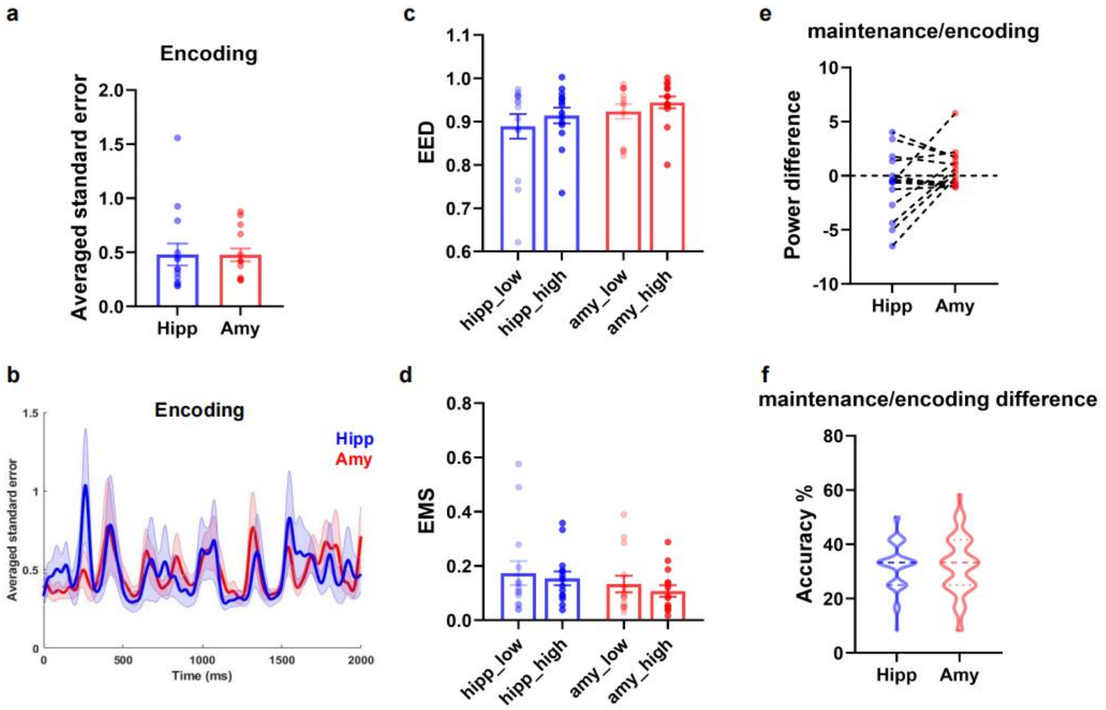

Figure S2 Control analyses. a Averaged variability of the power at 1-40 Hz during the encoding period across trials, in the hippocampus (blue, mean ± s.e.m.) and the amygdala (red, mean ± s.e.m.). Dots denote individual participants (n =14). No difference in the variability was found between the two regions. **b** The power variability (±s.e.m. shown as shading around the mean trace) across 1-40 Hz at each time point of the encoding period, in the hippocampus (blue) and the amygdala (red). No difference was found at each time point between the two regions. **c-d** encoding-encoding dissimilarity (EED) (**c**) and encoding-maintenance similarity (EMS) (**d**) in high-load trials (dark color) and low-load trials (light color) in the amygdala (red, mean ± s.e.m.) and hippocampus (blue, mean ± s.e.m.). No difference was found between high-load and low-load trials. Dots denote individual participants (n =14). **e** Relative difference of power indicating encoding/maintenance difference within the amygdala (red) and the hippocampus (blue) for each participant.

No difference was found between regions. **f** Decoding accuracy using the relative difference of power within the amygdala (red) and the hippocampus (blue). WM load could not be decoded by features from either region and no difference was found from all cross-validations (n =100) between regions. Dotted lines indicate the median. Broken lines above and below denote the quartiles. Source Data are provided as a Source Data file.

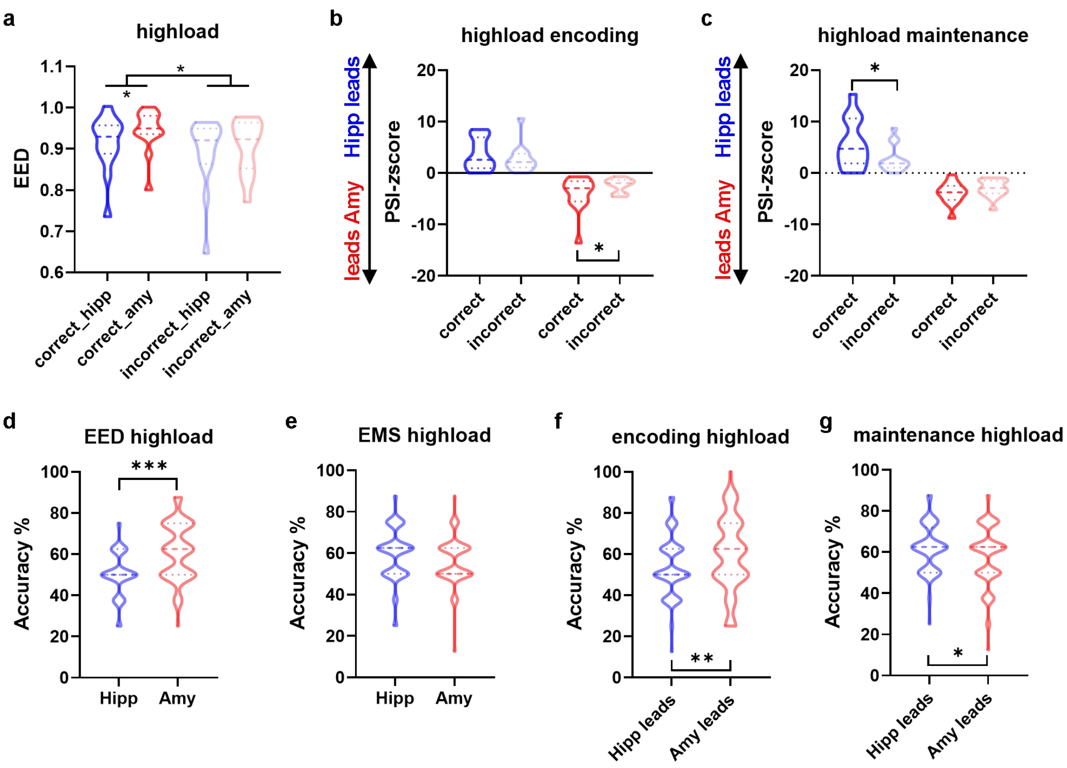

Figure S3. Success effects for the high load conditions and corresponding decoding accuracy within the amygdala and the hippocampus. a Encoding-encoding dissimilarity (EED) values in the amygdala (red) and the hippocampus (blue) for the correct (left) and incorrect trials (right), respectively. The EED values for the correct trials were higher than those for the incorrect trials (n =14 participants; repeated-measures ANOVA: p = 0.026, F(1,13) = 6.28). * $p < 0.05$. **b-c** Phase slope index (PSI) of the amygdala leads connectivity (red) and the hippocampal leads connectivity (blue) for the correct and incorrect trials during encoding (**b**) and maintenance (**c**), respectively. During encoding, the amygdala leads connectivity was larger (repeated-measures ANOVA: p = 0.042) at the correct trials than the incorrect trials while no difference was found from the opposite direction (n =14 participants; p = 0.21). During maintenance, the hippocampal leads connectivity was larger (repeated-measures ANOVA: p = 0.034) at the correct trials than the incorrect trials

while no difference was found from the opposite direction (n =14 participants; p = 0.14). * $p < 0.05$. **d** Using the features from the amygdala (red) was able to decode the WM performance with higher decoding accuracy than that from the hippocampus (blue) across cross-validations (n =100; two-sided permutation test, $p < 0.001$). *** $p < 0.001$. **e** Using the encoding-maintenance similarity (EMS) features from the hippocampus (blue) was able to decode the WM performance with higher decoding accuracy than that from the amygdala (red) across cross-validations (n =100; two-sided permutation test, $p = 0.062$). **f-g** Decoding accuracy using the PSI features from both directions during encoding (**f**) and maintenance (**g**). WM performance could be better decoded with the PSI features from the amygdala leads during encoding (n =100 cross-validations; two-sided permutation test, $p = 0.005$) and the PSI features from the hippocampus leads during maintenance (n =100 cross-validations; two-sided permutation test, $p = 0.025$). ** $p < 0.01$, * $p < 0.05$. Source Data are provided as a Source Data file.
