# Supplementary material for: Functional specialization and interaction in the amygdala-hippocampus circuit during working memory processing
Source: Nat Commun. 2023 May 22;14:2921. doi: 10.1038/s41467-023-38571-w (PMC10203226; doi:10.1038/s41467-023-38571-w)
Supplement: Supplementary file 3 — Reporting Summary [file 41467_2023_38571_MOESM3_ESM.pdf]

nature portfolio

Corresponding author(s): Tianzi Jiang, Johannes Sarnthein

Last updated by author(s): Apr 6, 2023

Reporting Summary

Nature Portfolio wishes to improve the reproducibility of the work that we publish. This form provides structure for consistency and transparency in reporting. For further information on Nature Portfolio policies, see our Editorial Policies and the Editorial Policy Checklist.

Statistics

For all statistical analyses, confirm that the following items are present in the figure legend, table legend, main text, or Methods section.

n/a	Confirmed	
☐	☒	The exact sample size (*n*) for each experimental group/condition, given as a discrete number and unit of measurement
☐	☒	A statement on whether measurements were taken from distinct samples or whether the same sample was measured repeatedly
☐	☒	The statistical test(s) used AND whether they are one- or two-sided<br>*Only common tests should be described solely by name; describe more complex techniques in the Methods section.*
☒	☐	A description of all covariates tested
☒	☐	A description of any assumptions or corrections, such as tests of normality and adjustment for multiple comparisons
☐	☒	A full description of the statistical parameters including central tendency (e.g. means) or other basic estimates (e.g. regression coefficient) AND variation (e.g. standard deviation) or associated estimates of uncertainty (e.g. confidence intervals)
☐	☒	For null hypothesis testing, the test statistic (e.g. *F*, *t*, *r*) with confidence intervals, effect sizes, degrees of freedom and *P* value noted<br>*Give P values as exact values whenever suitable.*
☒	☐	For Bayesian analysis, information on the choice of priors and Markov chain Monte Carlo settings
☒	☐	For hierarchical and complex designs, identification of the appropriate level for tests and full reporting of outcomes
☐	☒	Estimates of effect sizes (e.g. Cohen's *d*, Pearson's *r*), indicating how they were calculated

Our web collection on statistics for biologists contains articles on many of the points above.

Software and code

Policy information about availability of computer code

Data collection	Neurophysiological data were collected using the ATLAS system (Neuralynx Inc., Bozeman, Montana, USA).
Data analysis	Custom code (https://doi.org/10.5281/zenodo.7804834) written in MATLAB version R2018b (the Mathworks, Natick, MA, USA), FieldTrip functions (Fieldtrip Toolbox 20190527, https://www.fieldtriptoolbox.org/), and EEGLAB toolbox (version eeglab 14_1_0b, https://sccn.ucsd.edu/eeglab/index.php) were used to analyze all data. Anatomical data analysis was performed using Fieldtrip Toolbox 20190527.

For manuscripts utilizing custom algorithms or software that are central to the research but not yet described in published literature, software must be made available to editors and reviewers. We strongly encourage code deposition in a community repository (e.g. GitHub). See the Nature Portfolio guidelines for submitting code & software for further information.

Data

Policy information about availability of data

All manuscripts must include a data availability statement. This statement should provide the following information, where applicable:

- Accession codes, unique identifiers, or web links for publicly available datasets
- A description of any restrictions on data availability
- For clinical datasets or third party data, please ensure that the statement adheres to our policy

Data Availability. Source data are provided with this paper. The raw data used in this study (Boran et al. 2020, doi: 10.1038/s41597-020-0364-3) have been

downloaded from a public database under accession link https://doi.gin.g-node.org/10.12751/g-node.d76994/. The task is freely available for download at http://www.neurobs.com/ex_files/expt_view?id=266. Links to updates and further data sets can be found at https://hfozuri.ch.

Human research participants

Policy information about studies involving human research participants and Sex and Gender in Research.

Reporting on sex and gender	Sex and gender were not relevant variables in our analysis.
Population characteristics	14 participants (mean ± SD [range]: 34.5±12.6 [18-56]; 7 females) participated in this study.
Recruitment	We included all patients that accepted to participate in the study and that had electrodes implanted in the amygdala and hippocampus in the same hemisphere. Implantation sites were chosen solely on the basis of clinical criteria.
Ethics oversight	Before testing, all participants provided written informed consent for the study, which had been approved by the relevant institutional ethics review board (Kantonale Ethikkommission Zürich, PB 2016-02055).

Note that full information on the approval of the study protocol must also be provided in the manuscript.

Field-specific reporting

Please select the one below that is the best fit for your research. If you are not sure, read the appropriate sections before making your selection.

☐ Life sciences ☒ Behavioural & social sciences ☐ Ecological, evolutionary & environmental sciences

For a reference copy of the document with all sections, see nature.com/documents/nr-reporting-summary-flat.pdf

Behavioural & social sciences study design

All studies must disclose on these points even when the disclosure is negative.

Study description	Particpants replied IN or OUT in each trial while neurophysiological data were recorded from intracranial electrodes. We recorded performance accuracy and reaction time. While participants performed the task, we recorded the intracranial EEG (iEEG).
Research sample	Participants were patients with epilepsy where electrodes were implantated for presurgical evaluation. Implantation sites were chosen solely on the basis of clinical criteria. The iEEG data were downloaded from (Boran et al. 2020, doi: 10.1038/s41597-020-0364-3). We selected participants which had electrodes in the amygdala and hippocampus of the same hemisphere.
Sampling strategy	Our analysis is based on iEEG recorded from 14 participants. The participants performed sessions with 50 trials each, up to 6 sessions per participant. No statistical methods were used to pre-determine sample size. The sample size is larger or equal to those in previous publications (Zheng et al., 2017, Nat Commun, doi.org/10.1038/ncomms14413; Pacheco Estefan et al., 2019, Nat Commun, 10.1038/s41467-019-09569-0).
Data collection	Participants viewed the stimuli on a screen of a computer and replied with a button press on a keyboard. The task is freely available on http://www.neurobs.com/ex_files/expt_view?id=266. iEEG data were collected using the ATLAS system (Neuralynx Inc., Bozeman, Montana, USA). Only the participant and the researcher were present at data collection, neither was blinded to the experimental goals or the hypothesis or the study.
Timing	We collected data from consecutive patients.
Data exclusions	Only trials with artifacts in the iEEG were excluded as detailed in the previous study (Boran et al. 2020, doi: 10.1038/s41597-020-0364-3).
Non-participation	All consecutive patients participated in the task and no patients declined participation.
Randomization	For each trial, a new stimulus set was created randomly. All participants performed the same task.

Reporting for specific materials, systems and methods

We require information from authors about some types of materials, experimental systems and methods used in many studies. Here, indicate whether each material, system or method listed is relevant to your study. If you are not sure if a list item applies to your research, read the appropriate section before selecting a response.

Materials & experimental systems

n/a	Involved in the study	
☒	☐	Antibodies
☒	☐	Eukaryotic cell lines
☒	☐	Palaeontology and archaeology
☒	☐	Animals and other organisms
☒	☐	Clinical data
☒	☐	Dual use research of concern

Methods

n/a	Involved in the study	
☒	☐	ChIP-seq
☒	☐	Flow cytometry
☐	☒	MRI-based neuroimaging

Magnetic resonance imaging

Experimental design

Design type	Clinical protocol for structural scanning
Design specifications	We used structural scanning only for electrode contact localization
Behavioral performance measures	We did not record any behavioral measures as the structural scanning was used only for contact localization.

Acquisition

Imaging type(s)	Structural MRI and CT
Field strength	MRIs were acquired on a 3T
Sequence & imaging parameters	T1-weighted MRI
Area of acquisition	whole brain scanning

Diffusion MRI ☐ Used ☒ Not used

Preprocessing

Preprocessing software	Fieldtrip Toolbox (version 20190527, https://www.fieldtriptoolbox.org/)
Normalization	Data were normalized to MNI space
Normalization template	MNI
Noise and artifact removal	No noise or artifact removal was used.
Volume censoring	As we only used structural MRI, volume censoring was not used.

Statistical modeling & inference

Model type and settings	The structural scanning was used for electrode contact localization.
Effect(s) tested	No effects were tested.

Specify type of analysis: ☐ Whole brain ☐ ROI-based ☐ Both

Statistic type for inference (See Eklund et al. 2016)	We did not run any statistical test on MRI data.
Correction	We did not apply statistical corrections.

Models & analysis

n/a	Involved in the study	
☒	☐	Functional and/or effective connectivity
☒	☐	Graph analysis
☒	☐	Multivariate modeling or predictive analysis
